# Detection of sulfur mustard simulants using the microwave atmospheric pressure plasma optical emission spectroscopy method

**DOI:** 10.3389/fchem.2023.1173870

**Published:** 2023-06-02

**Authors:** Dexin Xu, Cong Li, Liu Yang, Wenchao Zhu, Bangdou Huang, Cheng Zhang, Tao Shao

**Affiliations:** ^1^ State Key Laboratory of NBC Protection for Civilian, Beijing, China; ^2^ Beijing International S&T Cooperation Base for Plasma Science and Energy Conversion, Institute of Electrical Engineering, Chinese Academy of Sciences, Beijing, China; ^3^ University of Chinese Academy of Sciences, Beijing, China

**Keywords:** atmospheric pressure plasma, optical emission spectroscopy, microwave plasma, toxic agents, trace detection

## Abstract

Sulfur mustard (SM) is one kind of highly toxic chemical warfare agent and easy to spread, while existing detection methods cannot fulfill the requirement of rapid response, good portability, and cost competitiveness at the same time. In this work, the microwave atmospheric pressure plasma optical emission spectroscopy (MW-APP-OES) method, taking the advantage of non-thermal equilibrium, high reactivity, and high purity of MW plasma, is developed to detect three kinds of SM simulants, i.e., 2-chloroethyl ethyl sulfide, dipropyl disulfide, and ethanethiol. Characteristic OES from both atom lines (C I and Cl I) and radical bands (CS, CH, and C_2_) is identified, confirming MW-APP-OES can preserve more information about target agents without full atomization. Gas flow rate and MW power are optimized to achieve the best analytical results. Good linearity is obtained from the calibration curve for the CS band (linear coefficients *R* ^2^ > 0.995) over a wide range of concentrations, and a limit of detection down to sub-ppm is achieved with response time on the order of second. With SM simulants as examples, the analytical results in this work indicate that MW-APP-OES is a promising method for real-time and in-site detection of chemical warfare agents.

## 1 Introduction

Even though the Convention on the Banning of Chemical Weapons (CWC) came into effect in 1997 (The Chemical Weapons Convention CWC, 1997), there still exists the risk of chemical warfare agents (CWAs) due to terrorist attack or leakage. Sulfur mustard (SM) is one kind of highly toxic blister CWA, and the maximum safe concentration–time (*Ct*) of SM vapor is 5 mg/min/m^3^ with a latency of 0–6 h (McNutt et al., 2020). SM has been widely spread over the world in war and induced millions of casualties in history (Szinicz, 2005; Tang and Loke, 2012). With a simple structure, SM is also relatively easy to synthesize, threatening social security (Devi, 2016).

As the most probable dispersion route of SM is via aerosol or vapor (Young et al., 2020), it is desirable to explore a sensitive and on-line gas-phase detection method with rapid response, good portability, and cost competitiveness. Nevertheless, existing traditional methods, including flame photometric detector (FID), ion mobility spectrometry (IMS), and gas chromatography–mass spectrometry (GC–MS), cannot fulfil these requirements at the same time (Cordell et al., 2007; Harris et al., 2011; McKelvie and Thurbide, 2019). Recently, numerous innovative strategies have been investigated and developed, such as surface-enhanced Raman spectroscopy (Xu et al., 2021), fluorescent probe (Feng et al., 2021), quantum dot sensor (Alev et al., 2022), quartz crystal microbalance (Lee et al., 2019), and atmospheric pressure plasma optical emission spectroscopy (APP-OES) (Broekaert and Siemens, 2004; Karanassios, 2004; Niu et al., 2021).

Among these emerging methods, APP-OES is based on the dissociation of target agents and excitation of fragments under the reactive APP environment, and OES is collected during radiative transition (de-excitation) processes (Niu et al., 2021). Distinguished from the traditional inductively coupled plasma (ICP) OES method, where plasma is at a nearly thermal equilibrium condition and the target agents are fully atomized, APP can be generated with non-equilibrium, called non-thermal APP (Shao et al., 2018). This indicates that the mean energy or temperature of mobile electrons with a small inertia is much higher than that of the background gas (Zhu et al., 2008). Benefitting from these characteristics, the target agents could be partially dissociated by non-thermal APP under a relatively low gas temperature, reserving more structure information about agents. Therefore, OES from both characteristic elements and radicals can be efficiently excited by high-energy electrons (Huang et al., 2023). It is also found that the OES intensity ratio of different radicals can be used to distinguish agents with similar element components, which further extends the discriminating ability of APP-OES (Yuan et al., 2012).

Up to now, many types of APP sources have been developed and used for the trace analysis, including (but not limited to) dielectric barrier discharge (DBD) (Meyer et al., 2012; Jiang et al., 2016; Han et al., 2018), glow discharge (Meng and Duan, 2015; Zhu et al., 2018), electrolyte cathode discharge (Yuan et al., 2021), mini-point discharge (Li et al., 2018; Yang et al., 2021), and microwave (MW) discharge (Pohl et al., 2008; Yuan et al., 2016; Borowska et al., 2019; Jung et al., 2019; Williams et al., 2019; Müller et al., 2020; Akhdhar et al., 2021). Compared with other APP sources, MW discharge has a relatively high power density (i.e., a strong dissociation ability) and a large reaction region (i.e., a long residence time for agents), and a highly purified reaction environment, eliminating any contamination due to the metal electrode, could be obtained, showing a promising future for trace detection of WCAs. It should be noted that, even though most MW sources engage rare gases or nitrogen as the carrier gas, an MW plasma source-based ¼-wavelength resonator with only ambient air as the carrier gas has been developed (Yu et al., 2023).

In our previous work, the OES characteristic of different APP excitation sources has been compared (Yang et al., 2022), and the analytical performance of the microwave atmospheric pressure plasma optical emission spectroscopy (MW-APP-OES) method for WCA simulants containing phosphorus and chlorine has been investigated (Li et al., 2022). In this work, the MW-APP-OES method is extended to detect three kinds of SM simulants, 2-chloroethyl ethyl sulfide (2-CEES), dipropyl disulfide, and ethanethiol. Characteristic OES from the C atom, CS radical, and Cl atom is identified. Good linearity is obtained from the calibration curve (*R* ^2^ > 0.995 for CS band), and a limit of detection (LOD) down to sub-ppm is achieved.

## 2 Materials and methods

### 2.1 Chemicals and reagents

SM simulants engaged in this work are 2-CEES (C_4_H_9_ClS, 97%, Macklin), dipropyl disulfide (C_6_H_14_S_2_, 99%, Innochem), and ethanethiol (C_2_H_6_S, 98%, Macklin). Original SM simulants are used in the experiment without any pre-treatment. High-purity argon (99.999%, Jinghui Gas, China) is used as the carrier gas for MW-APP.

### 2.2 MW-APP-OES system

**FIGURE 1:**
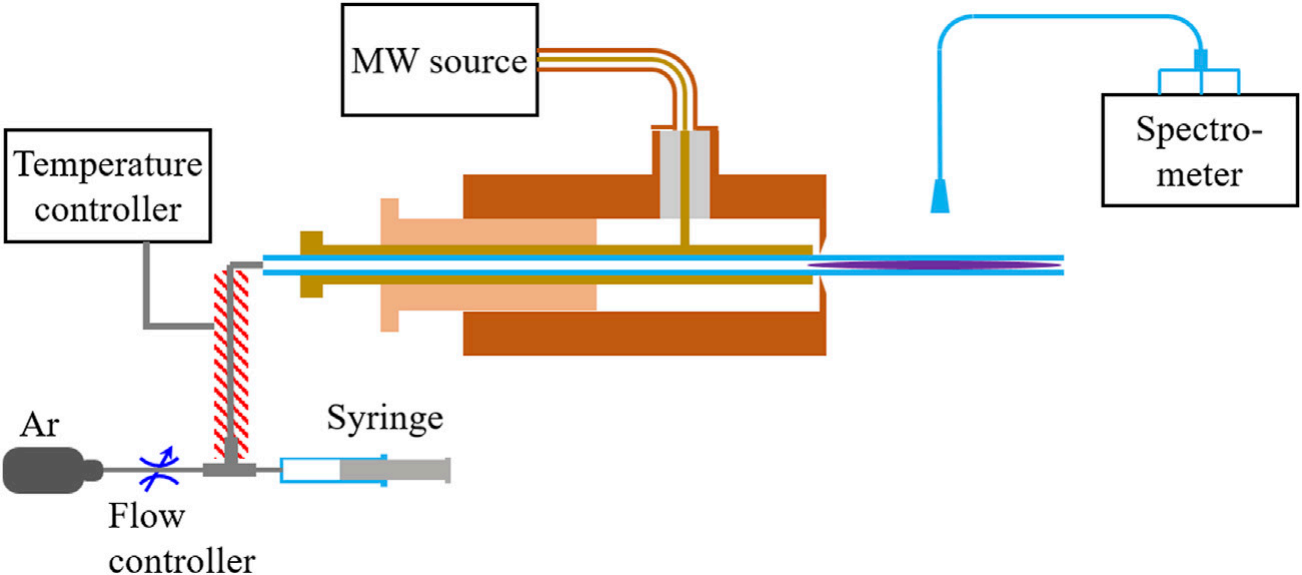
An illustrative diagram of the experimental setup.

Figure 1 shows an illustrative diagram of the MW-APP-OES system used in this work. An MW power source (WSPS-2450-200M, Wattsine, China) with a frequency of 2.45 GHz and a maximum power up to 200 W is used to generate MW-APP. A customized MW surfatron device is adapted to couple MW from the power source to plasma (Moisan and Nowakowska, 2018). Gas breakdown is induced at the slit of the surfatron, where MW electric field is locally intensified, and extended plasma is generated in a quartz tube with an inner diameter (ID) of 1.5 mm and an outer diameter (OD) of 3 mm. There are two tuning knobs in the MW surfatron, by rotating which the MW reflection from the plasma to power source can be reduced to below 5%.

A flow controller (G300C-5000sccm-P-24, Gas Tool Instrument, China) is used to control the rate of the carrier gas (argon). The gas line is heated, and its temperature is regulated using a temperature controller (AI-208FGL0, Udian, China). The SM simulants are injected into the gas line using a micro syringe (10 μL, Agilent), which is driven by a high-accuracy syringe pump (Pump 11 Elite, Harvard).

OES from MW-APP at around the middle position of the plasma column is collected by a fiber and is transferred to a three-channel spectrometer (PG2000-PRO-3, Ideaoptics, China), which covers a wavelength range of 196.79–1,039.58 nm (channel 1: 196.79–420.58 nm, channel 2: 406.59–623.60 nm, and channel 3: 604.89–1,039.58 nm) and provides a spectral resolution of about 0.1–0.2 nm.

### 2.3 Analysis procedure

When performing the MW-APP-OES analysis, the gas line is heated to a stable temperature of 180°C, which guarantees the full vaporization of SM simulants (in liquid phase at room temperature). The injection rate of SM simulants (*F*_s_) is controlled from 50–500 nL/min, via which their concentrations in the plasma environment are modulated. The flow rate of the carrier gas (*F*_Ar_) can be varied from 0.5–3.5 L/min, and the MW power can be varied from 50–120 W. The accumulation time of the spectrometer is fixed at 500 ms for channel 1, 200 ms for channel 2, and 100 ms for channel 3 in all measurements of this work.

As the gas line is heated to a relatively high temperature (180°C) and the injection rate of the target agent is very low, the vapor pressure of the target agent in the gas flow is much lower than its saturated value under the given temperature, and it is safe to assume the target agent is fully vaporized before arriving at the MW-APP region. Therefore, the concentration of target agent *C*_s_ (mg/m^3^) can be obtained from the ratio of the injection rate to the flow rate of the carrier gas (Li et al., 2022),

$$C_{s}=F_{s}∙\rho_{s}/F_{Ar},$$ (1)

and its volume concentration *C*_s_^V^ (ppm) is given by the following formula:

$$C_{s}^{V}=\left(F_{s}∙\rho_{s}/M_{s}\right)/\left(F_{Ar}/V_{m}\right).$$ (2)

Here, *ρ*_s_ and *M*_s_ are the density (g/cm^3^) and molar mass (g/mol) of the agent, respectively. *V*_m_ is the gas molar volume at STP (22.4 L).

## 3 Results and discussion

### 3.1 Characteristic OES of SM simulants

**FIGURE 2:**
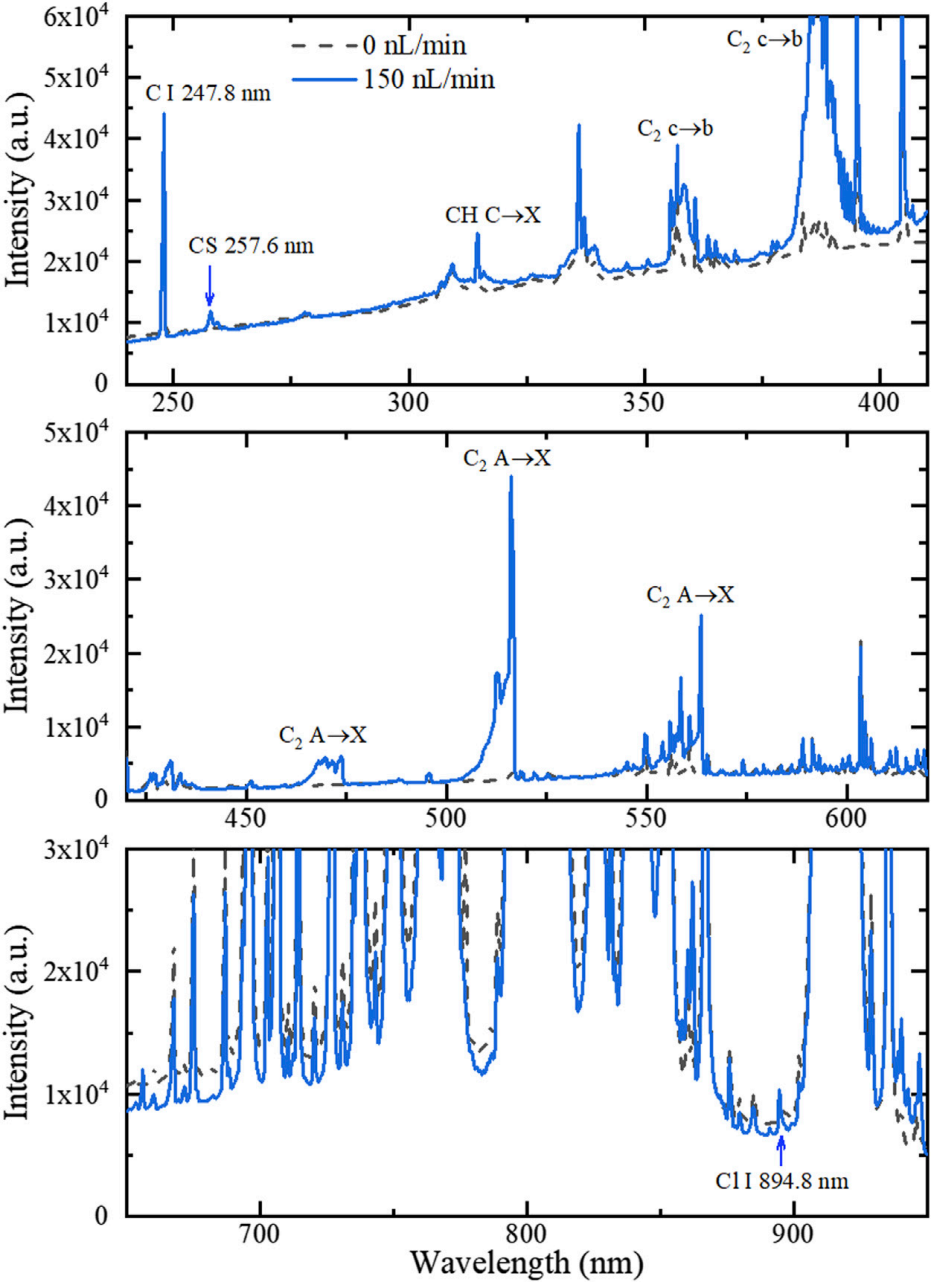
MW-APP-OES within different wavelength ranges for the blank and 2-CEES with an injection rate of 150 nL/min.

Figure 2 shows MW-APP-OES for the blank and 2-CEES with an injection rate of 150 nL/min.

By comparing these two groups of OES and looking into database (Gaydon and Pearse, 1963; NIST, 2022), it can be identified that the emerging OES after 2-CEES injection includes the C I line at 247.8 nm, CS band around 257.6 nm, CH C→X around 314 nm, C_2_ c→b band around 385 and 360 nm, and C_2_ A→X band around 516, 474, and 564 nm. These lines/bands can be clearly distinguished from the background. Furthermore, the Cl I line at 894.8 nm can also be identified, which is on the wind of argon lines, while most of other Cl I lines are overlapped with argon lines.

Based on the aforementioned observations, it can be analyzed that 2-CEES is partially dissociated in MW-APP, generating both atoms (C and Cl) and radials (CS, CH, and C_2_). As OES from C, CH, and C_2_ can be frequently observed in regular hydrocarbons (Meng and Duan, 2015), OES from CS and Cl should be particularly concerned when detecting 2-CEES. It should be noted that the CS band can also be frequently observed when detecting volatile organic sulfur compounds using OES excited by gas discharge (Li et al., 2018). Therefore, the Cl I line can be further involved to identify the agent. The characteristic OES from dipropyl disulfide and ethanethiol is similar to that of 2-CEES, except for the absence of Cl I, which will not be repeated here.

It is worth noting that the real samples containing target analytes can be mixed with interfering compounds at different concentrations producing similar or the same OES in the MW-APP. In this case, the target analyte cannot be identified and quantitatively analyzed solo by characteristic OES. To solve this challenge, coupling with a small gas chromatograph or ion mobility spectrometer as a front-end separation technique is one efficient way for this method to be applied in practical detection scenarios, which can solve the OES interference currently faced and the carrier gas issues.

### 3.2 Optimization of MW-APP-OES analysis

In order to obtain the best analytical results in MW-APP-OES quantitative analysis, plasma parameters, including gas flow rate and MW power, should be optimized.

**FIGURE 3:**
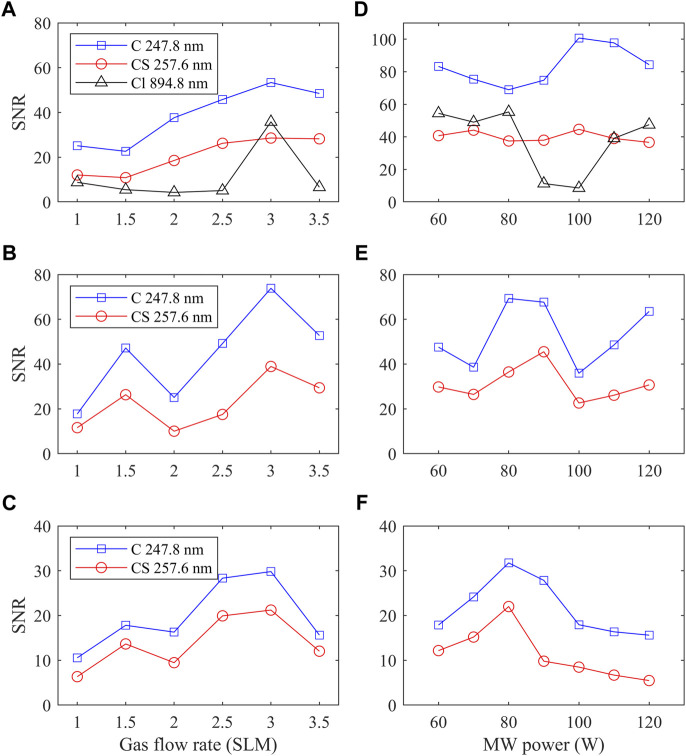
Optimization of the MW-APP-OES condition. The effect of the gas flow rate on the signal-to-noise ratio of the characteristic OES from **(A)** 2-CEES, **(B)** dipropyl disulfide, and **(C)** ethanethiol. **(D–F)** Effect of power on SNR for corresponding SM simulants.

Figures 3A–C show the effect of the gas flow rate on the signal-to-noise ratio (SNR) of the characteristic OES from 2-CEES, dipropyl disulfide, and ethanethiol, respectively. It should be noted that the injection rate of SM simulants is adjusted proportionally to the gas flow rate to maintain a constant concentration in the MW-APP environment. It can be observed that there is a peak for the relationship between SNR of the characteristic OES of all three SM simulants and the gas flow rate at around 3 SLM, which is chosen in the quantitative analysis below. This phenomenon is explained as follows: when the gas flow rate is small, the MW heating is not well taken away by the gas flow (i.e., overheating), which introduces instability for the MW plasma. On the contrary, when the gas flow rate is large, the gas flow in the quartz tube turns from laminar to turbulent, the latter of which also results in instability (Arnoult et al., 2008).

The effect of MW power on SNR is also explored for these three SM simulants (see Figures 3D–F). It can be seen that there is a peak for dipropyl disulfide and ethanethiol at around 80–90 W, which is not observed for 2-CEES. It should be noted that based on our previous investigation (Li et al., 2022), the OES intensity will increase almost linearly with MW power, while the signal-to-background ratio is nearly constant with MW power. In practice, one should balance the power consumption and OES intensity. In the quantitative analysis below, the MW power is set at 80 W.

### 3.3 Quantitative analysis and calibration curves

**FIGURE 4:**
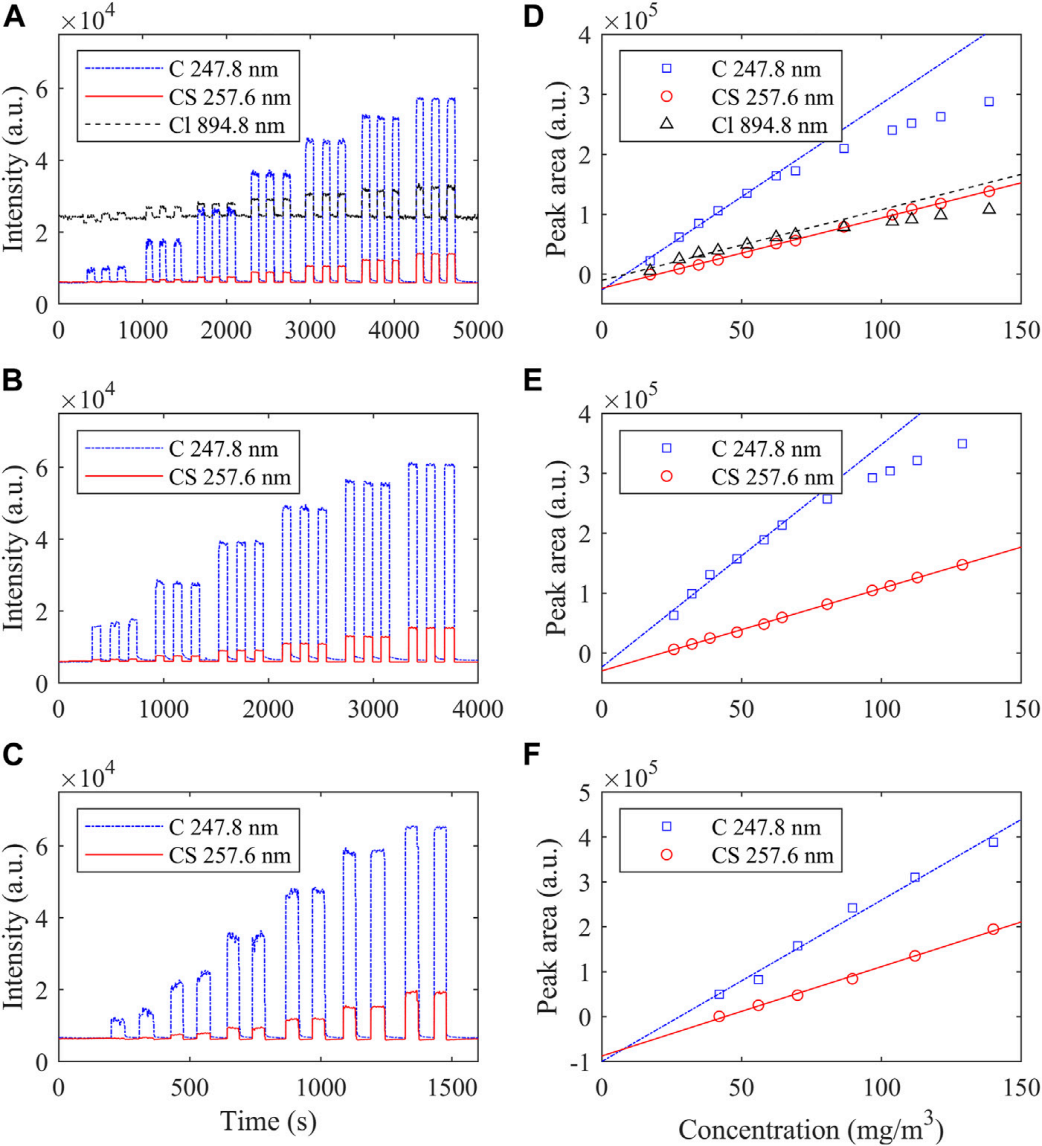
Temporal trace of MW-APP-OES of three SM simulations. **(A)** 2-CEES (*C*_s_ ∼ 17.3, 27.7, 41.6, 62.3, 86.6, 110.8, and 138.5 mg/m^3^), **(B)** dipropyl disulfide (*C*_s_ ∼ 25.8, 38.7, 58.0, 80.6, 103.1, and 128.9 mg/m^3^), and **(C)** ethanethiol (*C*_s_ ∼ 42.0, 50.3, 69.9, 89.5, 111.9, and 139.8 mg/m^3^). **(D–F)** Corresponding calibration curves.

Figure 4 shows the temporal trace of MW-APP-OES of three SM simulations, and the corresponding calibration curves with the analytical results are shown in Table 1.

**TABLE 1:** Analytical results of SM simulants by the MW-APP-OES method in this work.

SM simulant	Characteristic line/band	Fitting curve	*R* ^2^	LOD (mg/m^3^, ppm)	RSD (%)	Reproducibility (%)
2-CEES	C I 247.8 nm	*y* = 3.1 × 10^3^ *x*-2.6 × 10^4^	0.995	0.3, 0.05	1.9	4
	CS 257.6 nm	*y* = 1.2 × 10^3^ *x*-2.4 × 10^4^	0.999	1.5, 0.3	3.5	7
	Cl I 894.8 nm	*y* = 2.4 × 10^2^ *x*-2.1 × 10^3^	0.97	4.2, 0.8	2.9	7
Dipropyl disulfide	C I 247.8 nm	*y* = 3.7 × 10^3^ *x*-2.3 × 10^4^	0.986	0.3, 0.04	1.4	3
	CS 257.6 nm	*y* = 1.4 × 10^3^ *x*-3.0 × 10^4^	0.999	2.1, 0.3	2.6	6
Ethanethiol	C I 247.8 nm	*y* = 3.6 × 10^3^ *x*-1.0 × 10^4^	0.987	0.3, 0.1	3.4	2
	CS 257.6 nm	*y* = 2.0 × 10^3^ *x*-8.8 × 10^4^	0.996	1, 0.4	4.7	4

It can be seen that there is a clear square-wave feature on the temporal evolution of the OES intensity from characteristic line/band when the pulse injection of SM simulations is performed. The rising edge of OES intensity is as short as 1 s, indicating the rapid time response of the MW-APP-OES method in this work.

The intensity of CS 257.6 nm has a good linear relationship with the concentration of all three simulations from ∼20 to 140 mg/m^3^ (linear coefficients *R* ^2^ > 0.995). However, the intensity of C I 247.8 nm and Cl I 894.8 nm deviates from linearity when the concentration of 2-CEES is larger than ∼80 mg/m^3^. A similar phenomenon is also observed for dipropyl disulfide but does not exist for ethanethiol. The non-linearity at high concentrations is related to the partial dissociation and excitation processes of SM simulations in the MW-APP environment, and OES from radial bands shows better linearity compared with atom lines.

In order to quantify the analytical performance of the MW-APP-OES method, the LODs for three SM simulations are estimated by Shi et al. (2014)

$$LOD=3\sigma_{B}/k.$$ (3)

Here, *σ*_B_ is the standard deviation of characteristic OES intensity from 200 replicated blank cases, and *k* is the slope of the calibration curve. When the CS 257.6 nm band is used as the characteristic OES, the LODs of three SM simulations can reach a level as low as ∼ 1–2 mg/m^3^ (sub-ppm level).

In principle, the system developed in this work is tested using vapor of SM simulants. SM simulants are injected into the plasma environment with the carrier gas (argon) via the heated gas line for vaporization, and the characteristic OES is identified and analyzed. At the current stage, this system does not include a sampling scheme for gases (such as air) as the analyte.

Under actual testing conditions, the analytes are typically aerosols or vapors in the air. It would be necessary to develop an injection method to achieve carrier gas replacement, which can be achieved by collecting air samples with a given gas flow rate, adsorbing with active carbon, and desorbing the analytes using certain solvents and carrier gas, as has been performed by Jiang et al. (2016).

When carrier gas replacement is involved, the true LOD will be strongly influenced by the efficiency of adsorption and desorption processes and their time duration, i.e., how much analyte is gathered and released. As the current system only needs an analyte amount of several nL, based on the rise time of OES trace (i.e., response time) and injection rate, it may be safe to argue that when actual analytes in air are considered, an LOD similar to that using argon carrier gas (∼ppm) can still be obtained, and the LOD can further be extended with a longer adsorption time, sacrificing the response time.

The relative standard deviation (RSD) of the characteristic OES from three SM simulations is generally below ∼5%, obtained from the temporal trace of OES intensity during sample injection. Repeated experiments are also performed, and the reproducibility of the characteristic OES intensity from three SM simulations is generally better (∼10%) for concentrations larger than 50 mg/m^3^.

The recovery of the MW-APP-OES method is evaluated to confirm its accuracy. As there is no standard sample with known concentration for SM simulations, a recovery experiment is also performed using the original sample of SM simulants, and the concentration is controlled with the injection rate. A concentration of ∼50 mg/m^3^ is selected as the baseline, and more samples are added into the MW-APP environment. The CS 257.6 nm band is used as the characteristic OES, and the results are shown in Table 2. It can be seen that recoveries of this method for three SM simulations at different concentration levels are within the range of 80%–110%, indicating the utility of the current MW-APP-OES method.

**TABLE 2:** Recoveries of three SM simulants.

SM simulant	Added (mg/m^3^)	Found (mg/m^3^)	Recovery (%)
2-CEES	10.4	8.9	86
	17.3	13.9	80
	52.0	54.3	104
Dipropyl disulfide	9.7	8.6	89
	16.1	15.0	93
	48.4	50.0	103
Ethanethiol	14.0	14.3	102
	33.6	34.8	104
	83.9	90.6	108

Table 3 shows a comparison of the LOD and response time of different detection methods for 2-CEES. It can be seen that compared with the surface-enhanced Raman spectroscopy and fluorescent probe methods, which have a lower LOD, the MW-APP-OES method in this work has a much faster response time. Furthermore, expensive light sources in the former two methods are avoided in MW-APP-OES. Compared with the quartz crystal microbalance and quantum dot sensor methods, MW-APP-OES shows advantages in both LOD and response time.

**TABLE 3:** Comparison of the LOD and response time of different methods.

Method	Analyte	LOD	Response time	Reference
Surface-enhanced Raman spectroscopy	2-CEES	0.01 ppm	10 min	Xu et al. (2021)
Fluorescent probes	2-CEES	0.2 ppm	<4 min	Feng et al. (2021)
Quartz crystal microbalance	2-CEES	0.76 ppm	∼10 min	Alev et al. (2022)
Quantum dot sensor	2-CEES	0.5 ppm	3 s	Lee et al. (2019)
Liquid chromatography + DBD-OES + MW hydrolysis	Dithiocarbamate	0.1 μg/mL	>400 s	Han et al. (2018)
Photochemical vapor generation + miniaturized point discharge OES	Methylmercury	0.1 μg/L	∼10 s	Yang et al. (2021)
Dielectric barrier microhollow cathode discharge OES	Hydrochlorofluorocarbon	27 ppb	<1 s	Meyer et al. (2012)
DBD-OES	Dichloromethane	2 ng/mL	<1 s (15 min collection)	Jiang et al. (2016)
MW-APP-OES	2-CEES	1.5 mg/m^3^ or 0.3 ppm	1 s	This work

Table 3 also gives a comparison of the analytical performances of other APPs with the presenting method. As the target analyte, phase of the analyte (gas or liquid), and carrier gas in each APP are different, it is safe not to judge their performances simply. Compatibility with different carrier gases should be a key point in further investigations.

## 4 Conclusion

SM is one kind of highly toxic CWA and is easy to spread, threatening social security. However, existing traditional detection methods (FID, IMS, and GC-MC) cannot fulfil the requirement of rapid response, good portability, and cost competitiveness at the same time. In this work, the MW-APP-OES method is developed to detect three kinds of SM simulants, 2-CEES, dipropyl disulfide, and ethanethiol, taking the advantage of non-thermal equilibrium, high reactivity, and high purity of MW plasma. Characteristic OES from both atom lines (C I and Cl I) and radical bands (CS, CH, and C_2_) is identified, confirming MW-APP-OES can preserve more information about target agents without full atomization. The gas flow rate and MW power are optimized to achieve the best detection results. Good linearity is obtained from the calibration curve for the CS band (*R* ^2^ > 0.995) over a wide range of concentrations of SM simulations. A LOD down to sub-ppm is achieved with a response time on the order of second, showing competitiveness compared with other innovative detection methods to some extent. With SM simulants as examples, the analytical results in this work indicate that MW-APP-OES is a promising method for real-time and in-site detection of CWAs.

## Data Availability

The raw data supporting the conclusion of this article will be made available by the authors, without undue reservation.
